# Facilitators of professional psychological help-seeking among university students: a narrative review from an individual–environment dual-dimensional perspective

**DOI:** 10.3389/fpsyg.2026.1917476

**Published:** 2026-08-05

**Authors:** Chao Li

**Affiliations:** Henan Medical University, Xinxiang, Henan, China

**Keywords:** facilitators, narrative review, professional psychological help-seeking, theoretical integration framework, university students

## Abstract

The persistent disparity between the high prevalence of mental health problems among university students and their low rates of professional psychological help-seeking constitutes a well-documented “help-seeking paradox.” Research has primarily focused on barriers, while the systematic integration of facilitators remains underdeveloped. In particular, the field lacks a theoretical framework that can account for the dynamic interplay between individual and environmental factors. Using a narrative review approach, this article conducts a thematic analysis and theoretical synthesis of the extant literature. The findings indicate that key facilitators of professional help-seeking among university students can be organized into two overarching dimensions. Individual readiness encompasses three sub-dimensions (mental health literacy and help-seeking attitudes, emotional awareness and regulation, and personality traits and self-efficacy), and environmental driving force comprises three levels: the microsystem (family support and peer modeling), the mesosystem (campus culture and service accessibility), and the macrosystem (cultural values and media narratives). Building on this foundation, the review further examines the mechanisms underlying help-seeking initiation, moderation, and feedback, elucidating the synergistic enhancement or antagonistic inhibition that characterizes the interaction between individual readiness and environmental driving force. The proposed Individual Readiness–Environmental Driving Force integrative model conceptualizes professional help-seeking among university students as a process dynamically shaped by multilevel, multifactor influences, thereby providing a theoretical basis for developing systematic mental health intervention strategies for higher education settings.

## 1 Introduction

University students are at a critical stage of psychosocial development, confronting multiple stressors including academic demands, interpersonal relationships, and career planning; they therefore constitute a population at heightened risk for developing mental health problems (Juszczyk-Kalina et al., 2023). Research consistently demonstrates elevated prevalence of depression, anxiety, and other forms of psychological distress among this population (Alotaibi et al., 2024). Despite the pervasiveness of psychological distress, the proportion of students who actively seek professional help remains persistently low—this discrepancy between high need and low help-seeking is termed the “help-seeking paradox” (Ning et al., 2022; Wu et al., 2025). This phenomenon is ubiquitous worldwide: multiple cross-national studies report that the proportion of university students who receive professional mental health services is substantially lower than the proportion in need of them (Guenthner et al., 2023; Ning et al., 2024). For instance, a survey of university students in Hong Kong found that only one-quarter of those reporting moderate to severe depressive symptoms had sought professional help (Sum et al., 2024); a study on mental health service utilization following a campus mass shooting in the United States found similarly low utilization rates, even among students exhibiting posttraumatic stress and depressive symptoms (Reffi et al., 2022).

Existing research on university students’ help-seeking behaviors has primarily focused on barriers, such as mental illness stigma (Carvalho et al., 2024; Singh et al., 2023), misconceptions and distrust of psychological counseling (Ning et al., 2022), low mental health literacy (Gorczynski and Sims-Schouten, 2024), and structural obstacles such as insufficient service accessibility (Pinho et al., 2025). Conversely, systematic research on facilitators remains relatively underdeveloped. While prior studies have identified several facilitating variables, including positive help-seeking attitudes, higher mental health literacy (Baklola et al., 2024; Hannan and Wells, 2023), and robust social support (Yang et al., 2024), these findings are scattered across diverse research contexts and target populations. How these factors interrelate and jointly motivate help-seeking behavior has yet to be systematically integrated or clearly articulated. This theoretical gap limits both a comprehensive understanding of help-seeking mechanisms and the evidence-based design of intervention programs. The present article adopts a narrative review approach and aims to answer the following three core questions: What are the key factors that facilitate professional psychological help-seeking among university students? How do these factors operate at different levels (individual, interpersonal, systemic)? How do individual and environmental factors jointly influence help-seeking behavior? The review draws on three complementary conceptual tools—the Theory of Planned Behavior (TPB) and Social Cognitive Theory (SCT), which center on individual decision-making and self-efficacy beliefs, and the socio-ecological model, which provides a multilevel environmental analysis framework. Building on this integrative theoretical framework, the review systematically synthesizes various facilitating factors to construct an analytical model encompassing the two dimensions of individual readiness and environmental driving force, embedded within mechanisms of help-seeking initiation, moderation, and feedback, in order to provide a theoretical basis and practical insights for the design of more targeted and systematic mental health intervention strategies for higher education settings.

## 2 Literature search and screening strategy

This review employed a comprehensive literature search strategy designed to identify and theoretically integrate studies on the facilitators of professional psychological help-seeking among university students. Given the marked heterogeneity in research designs, measurement instruments, and outcome assessments across this field, a narrative synthesis approach is most appropriate. Narrative review is better suited for the critical reading, thematic integration, and generation of conceptual insights across methodologically heterogeneous literatures.

### 2.1 Search strategy

A comprehensive search was conducted across PubMed, PsycINFO, and Web of Science. Both controlled vocabulary terms and natural-language keywords were employed to enhance sensitivity. Search terms included: (“professional psychological help-seeking” OR “psychological counseling” OR “mental health service utilization”) AND (“college student*” OR “university students” OR “higher education”) AND (“mental health” OR “depression” OR “anxiety” OR “stress” OR “psychological distress”) AND (“facilitating factors” OR “enablers” OR “help-seeking attitudes”). The asterisk (*) was used as a wildcard to expand search terms where applicable. Since this review’s core research questions focus on university students, search terms centered on university student terminology were used. To incorporate relevant adolescent studies, the inclusion criteria were extended to the 14–25-year age range, which corresponds to the developmental period from late adolescence to emerging adulthood. This broader scope was intentionally adopted to capture studies that can illuminate developmental precursors of help-seeking attitudes and behaviors, precursors that are likely to persist and manifest throughout the university years. The search was limited to peer-reviewed literature published in English between January 2000 and March 2026. This timeframe spans the empirical accumulation period between Jorm's (2000) introduction of the mental health literacy construct and the present day. The reference lists of included studies and relevant review articles were also manually screened to capture relevant studies potentially missed during the database searches.

### 2.2 Inclusion and exclusion criteria

Inclusion criteria: (1) study participants were adolescents and university students aged 14–25; (2) the study addressed facilitators of professional psychological help-seeking; (3) the study employed quantitative, qualitative, or mixed-methods empirical designs, or was a review with clearly stated research questions and a systematic search strategy; (4) the study was published in a peer-reviewed journal. Exclusion criteria: (1) participants outside the 14–25 age range (i.e., children or non-student adult populations); (2) studies focusing solely on barriers to help-seeking without addressing facilitators; (3) studies that lacked empirical data or had questionable methodological quality.

### 2.3 Search and screening results

The initial literature search yielded 714 records from PubMed, 228 from PsycINFO, and 343 from Web of Science, for a total of 1,285 records across all electronic databases. Manual screening of reference lists identified an additional five records. After removing duplicates and reviewing titles, abstracts, and full texts, 72 studies met the inclusion criteria and were included in this review.

## 3 Theoretical framework

The theoretical integration in this review draws on three complementary theoretical perspectives: the TPB (Ajzen, 1991), SCT (Bandura, 1986, 1997), and the socio-ecological model (Bronfenbrenner, 1979, 2005). The TPB positions behavioral intention as the immediate antecedent of help-seeking behavior and views intention as being shaped by three components—attitudes, subjective norms, and perceived behavioral control. This theory provides a structured framework for understanding help-seeking decisions at the individual level. However, the TPB’s cognitive orientation is limited in its ability to fully account for the emotional complexity of help-seeking and the multilayered nature of environmental influence. SCT, centered on self-efficacy beliefs, emphasizes the triadic reciprocal determinism among personal factors, behavior, and environment, thereby compensating for the TPB’s static analytical limitations. However, it fails to systematically address the hierarchical structuring of environmental influence. While the socio-ecological model partitions the environment into the microsystem (e.g., family, peers), mesosystem (e.g., university organizations), and macrosystem (e.g., cultural values and social norms), offering a spatial framework for understanding multilevel environmental influences, it lacks a microlevel account of individual psychological mechanisms. None of these theories alone is sufficient to fully capture the complexity of facilitators of university students’ professional help-seeking; by integrating them, however, they can simultaneously accommodate individual cognitive and affective processes along with the dynamic person–environment interplay, thereby providing theoretical support for the subsequent literature synthesis and model construction in this review.

## 4 Individual readiness: the internal foundation for professional help-seeking

Individual readiness refers to the internal conditions at the cognitive, affective, and personality levels that predispose university students to proactively seek professional psychological help. This review identifies three core sub-dimensions: mental health literacy and help-seeking attitudes, emotional awareness and regulation, and personality traits and self-efficacy.

### 4.1 Mental health literacy and help-seeking attitudes

Mental health literacy (MHL) refers to an individual’s ability to recognize mental disorders, understand their risk factors and causes, and their awareness of self-help and professional help-seeking pathways (Jorm, 2000). Empirical research demonstrates that MHL directly influences individuals’ recognition of psychological problems and their subsequent help-seeking decisions (Chao et al., 2020; Shi and Tian, 2025; Trompeter et al., 2022). University students with higher MHL are more inclined to view professional psychological help as an effective solution; a study of undergraduate pharmacy students in Saudi Arabia found a significant positive correlation between MHL and help-seeking behavior (r = 0.26, *p* < 0.01; Almanasef, 2021), and other research revealed that students with a formal diagnosis of depression or who reported more severe depressive symptoms were more likely to use mental health services (Raposa et al., 2025).

Recognizing a problem does not necessarily lead to help-seeking behavior, however. For instance, a study of nursing students found that despite high detection rates for depression (30%), anxiety (38%), and posttraumatic stress (30%), symptom severity was not significantly associated with actual mental health service utilization (Jardon and Choi, 2024). This indicates a cognitive gap between “recognizing psychological distress” and “perceiving a need for professional intervention.” Thus, systematic mental health education should go beyond knowledge transmission and aim to strengthen students’ beliefs in the effectiveness of professional help-seeking, thereby laying the cognitive foundation for facilitating help-seeking behavior. Holding positive attitudes toward professional psychological help is another key driver of help-seeking intentions, with research demonstrating a close association between help-seeking behavior and attitudes toward seeking professional psychological help (El-Hachem et al., 2023; Li et al., 2025).

Students’ trust in the competence of psychological counseling practitioners also notably predicts their intentions to seek help (Chen et al., 2022). A qualitative study of Asian American university students found that favorable perceptions of the professionalism and confidentiality of campus mental health services enhanced students’ positive expectations of the counseling process (Dong et al., 2022). Empirical research further confirms that an individual’s perceived benefits of help-seeking constitute a core predictor of behavioral intentions (Wang et al., 2024), and holding positive beliefs about mental health malleability—that is, believing that psychological states can be improved through effort and intervention—positively predicts help-seeking intentions (Chen and Dang, 2025).

Reducing stigma is central to eliminating barriers and cultivating an environment that facilitates professional help-seeking. Self-stigma has been identified as the strongest negative predictor of help-seeking attitudes (Kim and Castillo, 2025), while perceived public stigma is also significantly negatively correlated with help-seeking attitudes (Wang et al., 2025). Multiple intervention studies have demonstrated that integrating mental health knowledge education with contact experiences with individuals in recovery can effectively reduce stigma. For example, the “ARTEMIS” education-contact intervention in Singapore significantly improved university students’ ability to identify depression, while also reducing social distance and personal stigma (Subramaniam et al., 2020); the “Transitions” intervention in Canadian postsecondary institutions, which provided mental health knowledge and life skills training, significantly improved participants’ mental health knowledge and reduced stigma (Wei et al., 2022). Intervention strategies centered on knowledge education and contact experiences therefore represent effective pathways for transforming stigma from a barrier into a facilitator.

### 4.2 Emotional awareness and regulation

Emotional awareness indirectly affects professional help-seeking decision-making by influencing individuals’ appraisal of the severity of psychological distress. When individuals have greater emotional awareness, they can recognize their own psychological distress earlier and more accurately (Jing et al., 2022). Perceived severity of psychological distress—the recognition that distress affects academic, interpersonal, or daily functioning—is a key cognitive prerequisite for help-seeking behavior (Wang et al., 2024). However, recognizing distress is merely a necessary condition; individuals must also judge that the distress exceeds their self-coping capacity before a decision to seek professional help can take shape. This judgment process is profoundly shaped by attributional patterns. When individuals attribute psychological distress to internal deficiencies such as lack of willpower, the likelihood of seeking professional help is significantly reduced (Fekih-Romdhane et al., 2021). Students with well-developed emotion regulation skills are more likely to view seeking professional psychological help as a proactive coping strategy. Research indicates that individuals’ beliefs about emotional controllability—believing that emotions can be regulated and changed through personal effort—profoundly shape emotion regulation strategy selection and mental health outcomes (Moumne et al., 2021, 2024). A systematic review further confirmed that higher emotion controllability beliefs are associated with lower levels of anxiety and depression and a greater likelihood of adopting adaptive emotion regulation strategies (Somerville et al., 2024). Intervention strategies targeting emotion regulation skills can foster more positive coping attitudes. One study found that the serious-game-based “emoWELL” intervention effectively improved participants’ emotion regulation skills and indirectly promoted self-acceptance and environmental mastery (Velert-Jiménez et al., 2025). Overall, systematically enhancing university students’ emotional awareness and emotion regulation skills offers a key pathway for strengthening their help-seeking readiness at affective and cognitive levels.

### 4.3 Personality traits and self-efficacy

Personality traits provide a stable dispositional foundation for university students’ professional help-seeking behavior. Individuals high in openness to experience are typically characterized by curiosity and a willingness to engage with new experiences. Research shows that high openness is associated with the adoption of problem-focused coping strategies when facing stress (Agbaria and Mokh, 2022). During the COVID-19 pandemic, Italian university students with high openness more frequently employed adaptive coping strategies, including proactive information seeking (Burro et al., 2023). This problem-oriented coping method makes individuals high in openness more likely to view professional psychological help-seeking as a problem-solving approach that is worth trying.

Extraversion may indirectly influence help-seeking behavior through social interaction. Extraverted individuals tend to have relatively richer social networks that may provide broader channels for obtaining mental health information and encouragement to seek help, thereby indirectly promoting their motivation for professional help-seeking. Self-efficacy—an individual’s belief in their capacity to accomplish specific tasks—is a core variable influencing help-seeking behavior. SCT posits that self-efficacy shapes individuals’ strategy choices and persistence in the face of difficulties. Research shows that self-efficacy mediates the relationship between shyness and help-seeking behavior, with low self-efficacy serving as a key mechanism inhibiting help-seeking among shy individuals (Patricio et al., 2025). Supportive communicative environments enhance students’ self-efficacy: a study grounded in SCT showed that family support and patient-centered communication from healthcare providers increased help-seeking readiness by increasing self-efficacy (Wu and Street, 2025). Research based on a modified TPB also confirmed that coping efficacy is a significant antecedent predicting help-seeking intentions (Pan and Hao, 2023). Cultivating an open cognitive style and a problem-oriented coping orientation, along with systematically enhancing self-efficacy within campus environments, can strengthen university students’ internal readiness to seek professional help at the levels of personality and beliefs.

## 5 Environmental driving force: the external empowerment system for professional help-seeking

Environmental driving force refers to the external conditions within an individual’s multilayered environmental systems that can facilitate or reinforce their propensity for professional psychological help-seeking. Drawing on Bronfenbrenner’s (1979, 2005) ecological systems theory, this review synthesizes these forces at three levels: the microsystem, mesosystem, and macrosystem.

### 5.1 Microsystem: family support and peer influence

Social support represents an important protective factor for university students’ mental health (Guo et al., 2021; Huang et al., 2021). Within the microsystem, family and peers are the most immediate interpersonal environment for university students, jointly shaping help-seeking behavior through two pathways: emotional foundation and behavioral norms. As family support constitutes the initial environment of individual socialization, its quality profoundly influences students’ help-seeking readiness when experiencing psychological distress. Research has shown that parenting styles are significantly associated with emotional distress among Chinese university students (Hou et al., 2020), and that parental emotional warmth helps individuals accumulate positive psychological resources (Xin, 2023). Conversely, family environments that are lacking in emotional support or characterized by severe conflict may increase adolescents’ risk for developing depression (Feng et al., 2025). Depression and anxiety among Slovenian postsecondary students during the pandemic were differentially associated with help-seeking depending on social support (Selak et al., 2024). Family emotional support not only serves as a protective factor for mental health but may also be an important mechanism shaping university students’ positive help-seeking attitudes and self-efficacy. Peers exert a more direct influence on university students’ professional help-seeking behavior; their destigmatizing attitudes toward mental health problems help reduce individuals’ self-stigma, and when peers treat psychological distress as a solvable health issue similar to physical health, individuals are more likely to internalize this normalizing perception, thereby reducing help-seeking-related shame (Kim and Hong, 2021; Khan et al., 2026). Peers’ help-seeking behavior also exerts a modeling effect. An intervention study of UK undergraduates found that the social-media-based “CoMUni” project, which shared students’ help-seeking experiences, effectively promoted participants’ help-seeking intentions (Wilde et al., 2025). Additionally, emotional support provided by peers provides a psychologically safe foundation for help-seeking. Peer support has positive effects on a range of mental health outcomes among adolescents and young adults (Richard et al., 2022). Peers’ destigmatizing attitudes, behavioral modeling, and emotional support work synergistically to jointly construct a peer culture that encourages professional help-seeking.

### 5.2 Mesosystem: campus culture and service accessibility

Campus mental health culture plays a central ecological role in shaping students’ help-seeking behavior. A study of 23,202 university students found that campus mental health climate significantly influenced help-seeking attitudes through the dual mediating pathways of reducing self-stigma and enhancing positive outcome beliefs (Lautenschlager and Zhou, 2025). Longitudinal research has further demonstrated that improvements in students’ perceived campus mental health culture were significantly associated with subsequent reductions in psychological distress and enhanced help-seeking intentions (Dunbar et al., 2025).

Regarding service formats, digital mental health interventions provide an important complement to traditional face-to-face counseling. A systematic review and meta-analysis found that digital mental health interventions have a moderate effect in alleviating mental health problems among university students (Madrid-Cagigal et al., 2025). The “Mind Booster Green” app-based cognitive behavioral therapy self-help intervention, which employs personalized narratives and gamification incentives, demonstrated the potential to reduce depressive symptoms among university students (Kim et al., 2025). Service accessibility—physical and psychological—is another critical factor influencing service utilization. Regarding physical accessibility, the low-cost nature of on-campus counseling services significantly lowers the economic threshold to seeking professional help (Peterson et al., 2025; Van Doren et al., 2024), and telemental health services overcome geographical barriers through online booking and remote counseling (Gatdula et al., 2024). Psychological accessibility concerns the extent to which service design aligns with students’ psychological characteristics. Services that are culturally adapted for specific groups can effectively reduce perceived psychological distance: research has found that LGB + university students had higher mental health service utilization rates than their heterosexual peers, indicating the positive role of an inclusive campus service environment (Bourdon et al., 2021). In addition to providing students with a psychologically safe space that does not require identity disclosure, anonymous service formats are particularly suitable for students who hesitate to seek help due to fear of stigmatization (Harris, 2024; Mehrotra et al., 2025). Mental health education and institutional-level support jointly constitute the superstructure of the campus psychological service ecosystem. Research has reported that nursing students who completed a semester-long psychiatric course exhibited improved attitudes toward seeking professional psychological help, suggesting that integrating mental health content into professional curricula may positively influence help-seeking attitudes (Abuhammad and Hamaideh, 2021). Public endorsement by university leadership, in turn, signals the institution’s commitment to mental health, indirectly lowering students’ perceived barriers to help-seeking (Dong et al., 2022).

### 5.3 Macrosystem: sociocultural context

Bronfenbrenner’s macrosystem refers to the overarching cultural, social-normative, and value systems within which individuals are embedded. For university students’ professional help-seeking behavior, macrosystems exert influence primarily through two pathways: first, by shaping individuals’ internal cognitions about psychological distress and help-seeking behavior through cultural values, and second, by influencing the wider societal mental health discourse through the education system and mass media. The influence of cultural values on help-seeking behavior is particularly profound. Within Chinese cultural contexts, the emphasis on social conformity and the concept of “face,” rooted in Confucian traditions, may incline individuals to associate mental illness with personal weakness or family shame and thereby internalize help-seeking-related public stigma (Wang et al., 2025). A survey of Tunisian university students found that 43.8% attributed mental illness to “lack of self-discipline and willpower” (Fekih-Romdhane et al., 2021), indicating that such moralizing attributions are prevalent across multiple cultural traditions and constitute a cross-cultural cognitive barrier to help-seeking. Pathways for improving the broader cultural environment are multifaceted. Systematic mental health education at the institutional level can enhance public mental health literacy; for example, MHL curriculum interventions for undergraduate public health students have shown positive effects (Lai et al., 2022). At the discursive level, mass media narrative framing directly shapes societal perceptions of mental health, with evidence suggesting that when the media portray positive narratives of recovery and hope, they can exert a protective effect analogous to the “Papageno effect” (Arendt et al., 2025), suggesting that destigmatizing media narratives may foster a more supportive social discourse environment to facilitate help-seeking.

## 6 The initiation, moderation, and feedback of help-seeking behavior

### 6.1 The initiation of help-seeking: triggering, appraisal, and feasibility

Sometimes, students who possess relatively high help-seeking readiness, hold positive cognitive orientations, and have access to environmental resources still fail to translate their help-seeking intentions into actual behavior (Hardy et al., 2025; Wao et al., 2025; Zhao et al., 2025). The elements involved in this initiation process can be synthesized into three categories: triggering events, subjective appraisals, and feasibility factors. Help-seeking initiation typically begins with a triggering event, a situational change that transforms a vaguely held intention to seek help into a concrete decision. Such events may originate internally (e.g., acute onset of severe distress, cumulative functional breakdown) or externally (e.g., explicit encouragement from significant others; Lui et al., 2024). Their shared function is to shift the notion of professional help-seeking from an abstract possibility to an option warranting serious evaluation. A triggering event alone is insufficient to generate a stable intention to seek help. Individuals must also complete a set of subjective appraisals to establish such an intention. Two core appraisal dimensions are particularly important. The first is perceived severity of distress—when psychological distress accumulates to the point that it affects academic, interpersonal, or daily functioning, individuals are more likely to identify it as “a health issue requiring professional intervention” (Wang et al., 2024). The second is belief in help-seeking effectiveness—Zhao et al.’s (2025) meta-analysis further indicates that perceived severity and this belief jointly constitute key predictors of help-seeking intentions. When individuals’ appraisals of both dimensions reach a level sufficient to motivate help-seeking action, a necessity judgment is formed, and help-seeking intention emerges. While triggering events and subjective appraisals are conceptually distinguishable, they often occur simultaneously: individuals appraise the severity of their distress even as they experience triggering events. Note that the establishment of a necessity judgment does not automatically translate into actual help-seeking behavior; individuals must also appraise the real-world feasibility of seeking help, which involves dimensions such as time investment, economic burden, psychological concerns (worries about stigma, identity exposure anxiety), and physical accessibility (geographic distance, mobility convenience; Peterson et al., 2025; Van Doren et al., 2024).

### 6.2 The help-seeking moderation and feedback loop

The relationship between individual readiness and environmental driving force is not simply additive. When the environmental driving force is insufficient, the conversion efficiency of individual readiness into help-seeking behavior is markedly reduced; when individual readiness is insufficient, the utilization rate of environmental resources is likewise constrained. A systematic review and meta-analysis revealed that many students struggle to effectively utilize campus mental health services even when they are objectively available (Zhao et al., 2025). A systematic review of ethnic minority students found that their help-seeking intentions were significantly suppressed by structural barriers and cultural incongruence (Hardy et al., 2025). In resource-limited African regions, insufficient service accessibility is a key barrier to help-seeking (Wao et al., 2025). Therefore, when both forces are at relatively high levels, synergistic enhancement maximizes the probability of help-seeking; when either force is at a relatively low level, antagonistic inhibition may attenuate the facilitative effect of the other. The process of moderating psychological help-seeking behavior is not unidirectional or one-shot; rather, experience with help-seeking represents an important feedback loop within this process. Help-seeking experiences influence individuals’ self-efficacy and self-stigma, in turn shaping subsequent individual readiness and environmental perceptions. Westberg et al.'s (2025) qualitative study identified four types of healthcare journeys (smooth, fragmented, exploratory, and resource-limited), and Cerolini et al.'s (2023) systematic review emphasized the important role of the quality of counseling experiences in shaping students’ subsequent help-seeking decisions. This feedback characteristic suggests that help-seeking behavior follows a cyclical, dynamically evolving pattern—individual readiness and environmental driving forces are updated or consolidated through help-seeking experiences, which in turn influence the next round of help-seeking decisions.

## 7 Theoretical contributions and practical implications

### 7.1 Theoretical contributions

Based on a critical synthesis and theoretical integration of the existing literature, this review develops an analytical model that incorporates two dimensions—individual readiness and environmental driving force—and is embedded with mechanisms for help-seeking initiation, moderation, and feedback. Compared with prior research, this model’s principal strengths are threefold: first, the model shifts the perspective from “barriers” to “facilitators.” While prior reviews have predominantly focused on descriptive compilations of barriers such as stigma, low literacy, and service inaccessibility, the present review adopts a positive perspective, systematically extracting multidimensional factors that effectively facilitate help-seeking behavior, thereby providing positive targets for intervention design. Second, it transcends a single-dimensional analytical framework. Existing studies have largely examined influencing factors in isolation at the individual or environmental levels. By integrating the TPC, SCT, and the socio-ecological model, this review unifies these disparate factors into an Individual Readiness–Environmental Driving Force framework, elucidating the synergistic enhancement or antagonistic inhibition that characterizes the interaction between these two dimensions. Third, the present model proposes an analytical framework with a sharper focus on psychological help-seeking behavior, emphasizing the interplay across ecological system levels and incorporating mechanisms of help-seeking initiation, moderation, and feedback. This breaks through the limitations of static analysis and reveals the cyclical, dynamically evolving characteristics of help-seeking behavior, providing an analytical lens that is structurally and processually oriented for understanding university students’ professional help-seeking behavior.

### 7.2 Practical implications

Based on the integrative model, promoting university students’ professional help-seeking behavior requires coordinated interventions across three levels, individual, interpersonal, and institutional. At the individual level, mental health education should combine knowledge transmission with capacity building. For example, curriculum-based and promotional activities, including MHL modules during new student orientation, can enhance students’ ability to recognize psychological problems, their knowledge of help-seeking pathways, and their trust in the effectiveness of professional services. Concurrently, sharing successful help-seeking experiences can enhance students’ help-seeking self-efficacy, and training in emotion identification and regulation skills can strengthen their proactive strategies for coping with psychological distress. At the interpersonal and peer level, student leaders and academic advisors can serve as help-seeking facilitators. The destigmatizing attitudes of peers and their own help-seeking behaviors exert a modeling effect, while the timely guidance and encouragement provided by advisors constitute a key source of social support that helps students overcome the psychological threshold for help-seeking. Systematic training for these two roles—covering early recognition of psychological problems, supportive communication skills, and help-seeking resource navigation—should form an integral component of campus mental health promotion systems. Integrating culturally responsive approaches, such as offering counseling services in multiple languages and training staff in the cultural norms of diverse student populations, in light of the cultural variability in help-seeking facilitators. At the institutional level, mental health promotion should be embedded in campus culture and institutional design. Specific measures could include enhancing the physical (e.g., optimizing the geographic location of counseling centers) and psychological (e.g., providing anonymous counseling options, developing digital mental health tools, such as online self-screening and triage platforms that direct students to appropriate service levels based on symptom severity) accessibility of mental health services, securing sustained policy and budgetary support from leadership, and establishing effective mechanisms for service feedback and continuous quality improvement. All of these approaches could help facilitate university students’ professional help-seeking behavior.

### 7.3 Limitations and future directions

As a narrative review, the present study has several limitations. First, although we aimed for a comprehensive search, the processes of study screening and thematic synthesis inevitably rely on researchers’ subjective interpretation, and we did not apply a systematic scoring system to evaluate the methodological quality of each included study. Consequently, differences in design rigor among the studies may not have been equally weighted. Second, the included studies show considerable heterogeneity in research designs, sample characteristics, and measurement instruments, which may limit the comparability of findings across studies. Third, the review primarily identifies associative patterns among factors; inferring causal relationships would require longitudinal or experimental designs.

Furthermore, the evidence base for several facilitators reveals notable variability. The association between MHL and help-seeking intentions appears robust across diverse samples, but the link between symptom severity and actual service utilization is weaker, a discrepancy also evident in the nursing student study (Jardon and Choi, 2024). The conditions under which certain facilitators operate effectively remain insufficiently specified, for example, the cultural embeddedness of family support and peer influence may render findings from individualistic cultural contexts only partially generalizable to collectivistic settings.

Future research may advance in the following directions: first, longitudinal tracking designs should be employed to test the hypothesized causal relationships and dynamic interaction pathways in the present model, paying particular attention to how environmental factors shape individuals’ help-seeking developmental trajectories over time. Second, cross-cultural comparative studies should be conducted to examine the applicability of the model across different cultural contexts—in particular, how concerns regarding “face” and familistic collectivism interact with the model’s universal factors warrant dedicated exploration. Third, the composition of environmental driving force is evolving with the rapid advancement of artificial intelligence and digital mental health services; future research should therefore examine emerging factors such as digital literacy and trust in AI counseling. Fourth, multilevel intervention programs should be developed based on the integrative model, and their efficacy should be evaluated through designs such as randomized controlled trials, thereby advancing the translation of the theoretical framework into evidence-based practice.

## 8 Conclusion

**Figure 1:**
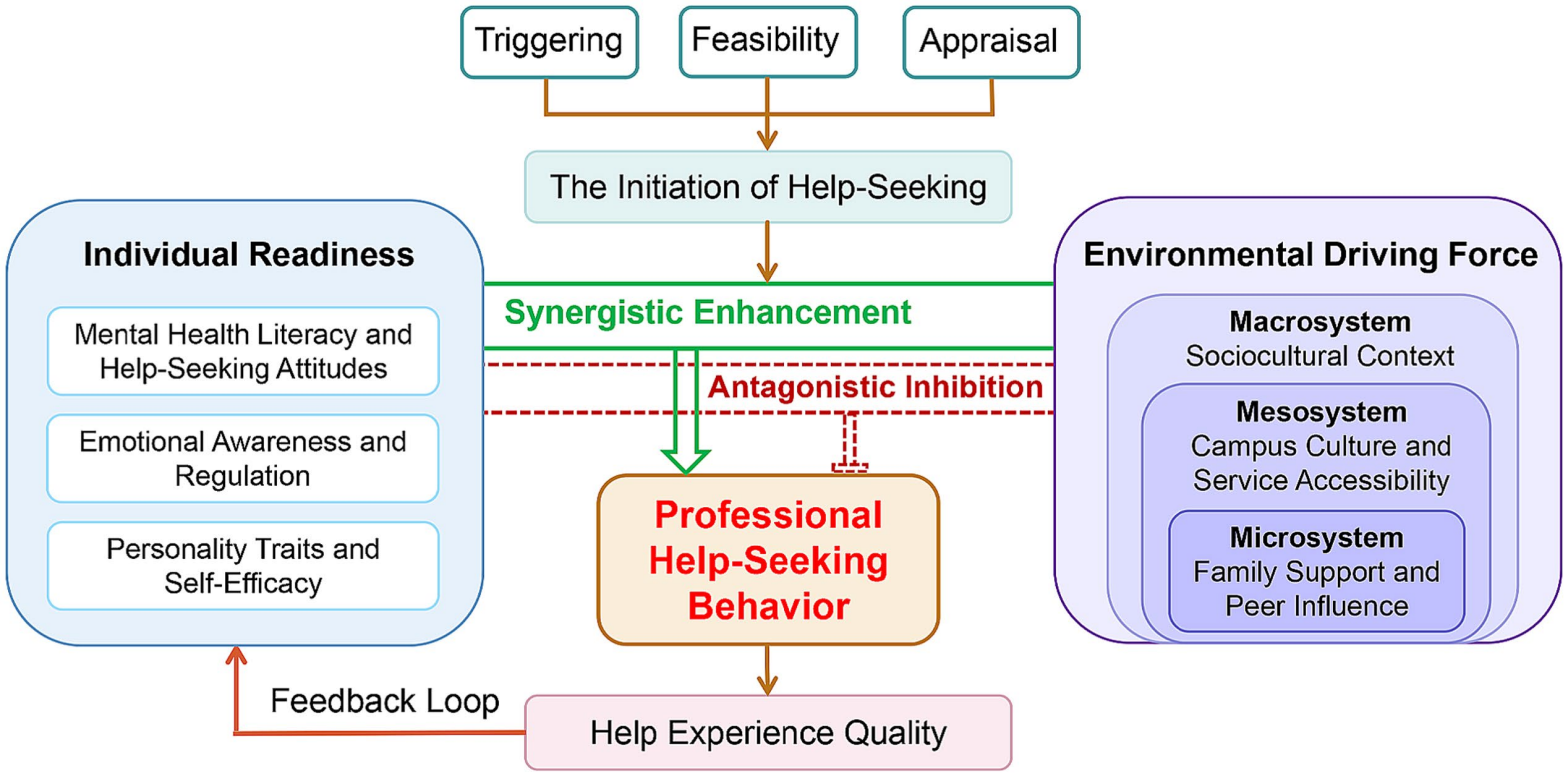
Individual Readiness––environmental driving forces integration theory model. This theoretical model delineates a multilevel interactive framework comprising two core dimensions (individual readiness and environmental driving forces), embedded with mechanisms of help-seeking initiation, moderation, and feedback loops. Individual readiness constitutes the internal foundation, whereas environmental driving forces offer external support. The interaction between the two dimensions, which functions through either synergistic enhancement or antagonistic inhibition, jointly determines the initiation and developmental trajectory of help-seeking behavior.

Using a narrative review approach, this article proposes a theoretical model that encompasses two dimensions—individual readiness and environmental driving force—and is embedded with mechanisms of help-seeking initiation, moderation, and feedback. The model’s core contribution lies in its integration of the factors that facilitate university students’ professional help-seeking behavior from isolated variables into a multilevel interactive system: Individual readiness constitutes the internal foundation, environmental driving force provides external support, and together they shape the emergence and development of help-seeking behavior through patterns of either synergistic enhancement or antagonistic inhibition (Figure 1). This systemic perspective provides a theoretical foundation for higher education institutions to overcome the “help-seeking paradox” and design intervention strategies that shift from isolated barrier elimination to bidirectional individual–environment empowerment.
